# The relationship between IL-10 levels, gene polymorphisms and histiocytic necrotizing lymphadenitis in children

**DOI:** 10.1186/s12887-026-06760-3

**Published:** 2026-04-15

**Authors:** Liyan Liu, Hualei Bai, Zhijie Xing, Jian Li

**Affiliations:** Department of Infectious Diseases, Children’s Hospital Affiliated to Shandong University (Jian Children’s Hospital), Jinan, Shandong China

**Keywords:** Histiocytic necrotizing lymphadenitis, Interleukin-10, Gene polymorphisms

## Abstract

**Background:**

It has been reported that interleukin-10 (IL-10) is associated with some immune-mediated diseases; however, its relationship with histiocytic necrotizing lymphadenitis (HNL) has not been reported. This study aimed to preliminarily investigate the relationship between IL-10 and HNL.

**Methods:**

This study included 35 children with HNL and 35 healthy controls. Plasma IL-10 levels were detected by ELISA. The gene polymorphisms at -1082A/G, -819T/C, and -592 A/C in the IL-10 promoter were analyzed by Sanger sequencing. Differences in IL-10 expression, allele and genotype frequencies between the two groups were statistically compared.

**Results:**

IL-10 levels were significantly higher in children with HNL than in the control group (*P* = 0.001). However, no significant differences were observed between the two groups in allele frequencies, genotype distributions of the three IL-10 promoter polymorphisms, or the haplotypes constituted by these loci.

**Conclusions:**

These findings suggest that IL-10 may contribute to HNL pathogenesis, while no significant association between the IL-10 promoter polymorphisms (-1082A/G, -819T/C, -592A/C) and pediatric HNL susceptibility was found in this exploratory analysis. A potential genetic association cannot be excluded and further studies with a larger sample size are warranted.

Histiocytic necrotizing lymphadenitis (HNL), also known as Kikuchi-Fujimoto disease, is a disorder characterized mainly by fever, cervical lymphadenopathy and leukopenia [1]. Although current studies generally attribute its pathogenesis to an excessive immune response triggered by pathogenic infections [2], the underlying mechanisms remain incompletely understood. Interleukin-10 (IL-10) is a key anti-inflammatory cytokine that plays a critical role in the negative regulation and timely termination of inflammatory processes [3]. The human IL-10 gene (Gene ID: 3586) is located on chromosome 1q32, and its promoter region contains several single nucleotide polymorphisms (SNPs), including -1082 A/G, -819T/C, and -592 A/C, which have been shown to influence IL-10 expression and thereby modulate immune function [4]. Previous studies have shown that the level of IL-10 and its genetic polymorphism are closely associated with some immune-mediated diseases such as systemic lupus erythematosus (SLE), asthma, and inflammatory bowel disease (IBD) [5–7]. However, the role of IL-10 in the pathogenesis of HNL has not been reported. This study aimed to investigate the relationship between IL-10 levels, IL-10 gene polymorphisms, and HNL in children.

## Subjects and methods

### Study population

This study included 35 children with HNL, who were hospitalized in our department between January 2023 and June 2025, as the case group. Patient enrollment and data collection were completed prior to analysis. The patients fulfilled all the following criteria: (1) recurrent fever and lymphadenopathy with a normal or low peripheral white blood cell count; (2) histopathological diagnosis of HNL via excisional lymph node biopsy; (3) exclusion of other etiologies including infectious, autoimmune, and malignant disorders. Patients were excluded for: (1) severe dysfunction of the heart, liver, or kidneys; (2) congenital malformations; (3) a history of medication with antineoplastic drugs, immunosuppressants, or anti-tuberculosis medications. Among the 35 cases, there were 24 males and 11 females, with a mean age of (10.06 ± 2.20) years. The control group consisted of 35 healthy children (21 males and 14 females; mean age, 10.20 ± 2.28 years), recruited from our outpatient department during routine health check-ups. All controls were free from infectious, autoimmune, or malignant diseases and had no recent history of immunosuppressant use. There were no statistically significant differences in gender and age between the two groups (*P* > 0.05). All children in both groups were Han Chinese. Written informed consent was obtained from the guardians of all participants. The study was approved by the Medical Ethics Committee of Children’s Hospital Affiliated to Shandong University (SDFE-IRB/P-2022053; July 2022) and conducted in accord with the Declaration of Helsinki.

### Clinical characteristics of patients and sample collection timing

All patients presented with fever and superficial lymphadenopathy, with moderate-to-high axillary temperatures ranging from 38.1 to 41 °C. Lymphadenopathy occurred most frequently in the cervical region; left axillary lymphadenopathy was identified in only one case. Most enlarged lymph nodes (31/35, 88.57%) were tender. Mild transaminase elevation and rash were each observed in two patients, with no other clinical manifestations noted. Following a definitive histopathological diagnosis, all patients with HNL received corticosteroid treatment. Complete resolution of clinical symptoms was achieved, and no complications were observed during follow-up.

Peripheral venous blood samples for IL-10 detection were collected from all patients during the acute phase of HNL and prior to the initiation of corticosteroid therapy. Relevant inflammatory markers, including peripheral white blood cell (WBC), Erythrocyte sedimentation rate (ESR), C-reactive protein (CRP) and procalcitonin (PCT), were measured by standard clinical laboratory methods within 24 h of sample collection.

### Measurement of IL-10 levels

Peripheral venous blood (2 mL) was collected from children in both groups and immediately placed in EDTA anticoagulant tubes to avoid hemolysis. All blood samples were transported to the laboratory within 1 h of collection. Plasma was separated by low-speed centrifugation (3000 r/min, 4 °C, 10 min), aliquoted into sterile 1.5-mL EP tubes, and stored at − 80 °C until subsequent analysis. Freeze-thaw cycles were strictly limited to no more than one to prevent protein denaturation. Plasma IL-10 levels were quantified by using a commercial enzyme-linked immunosorbent assay (ELISA) kit from MULTISCIENCES (LIANKE) BIOTECH, CO., LTD. (Hangzhou, China), strictly according to the manufacturer’s instructions.

For the human IL-10 ELISA, the kit exhibited a quantification range of 0.39–25 pg/mL, and a linear correlation coefficient greater than 0.99. The assay demonstrated high specificity, with no cross-reactivity observed with other cytokines, including IL-2, IL-6, TNF-α, and IFN-γ. To assess reproducibility, intra-assay and inter-assay coefficients of variation (CVs) were evaluated prior to formal experiments. The intra-assay CVs, determined by 10 repeated measurements of low, medium, and high-concentration quality control (QC) samples, were 2.1%, 1.8%, and 2.5%, respectively (all < 5%). The inter-assay CVs, calculated from three independent experimental batches with three replicates per batch, were 6.2%, 5.8%, and 7.1%, respectively (all < 10%). All experiments were conducted under strict quality control. Each sample was tested in duplicate, and the relative deviation of absorbance values between duplicate wells was required to be < 5%; the average value was used as the final result. Each 96-well plate included blank, negative control, and positive control wells. In addition, three QC samples at concentrations of 1 pg/mL, 5 pg/mL, and 20 pg/mL were inserted at regular intervals. If the optical density (OD) values of these QC samples deviated from the calibrated range by more than ± 2 standard deviations, the entire plate was discarded and repeated to ensure the accuracy of all measurements.

### Analysis of IL-10 gene promoter polymorphism

Genomic DNA was extracted using a genomic DNA extraction kit (Tiangen Biotech Co., Ltd., Beijing, China) in strict accordance with the manufacturer’s instructions. Specific primers for target loci were designed with Primer-BLAST software. For the − 1082A/G locus, the forward primer was 5’-GTAGAGCAACACTCCTCGCC-3’ and the reverse primer was 5’-GTAGGGGTCATGGTGAGCAC-3’, amplifying a target fragment of 450 bp. A common set of primers was used for the − 819T/C and − 592A/C loci: forward 5’-CCAGCCACAGAAGCTTACAAC-3’ and reverse 5’-GTCTTGGGTATTCATCCCAGGT-3’, with an amplified target fragment of 533 bp. All primers were synthesized by Tsingke Biotech Co., Ltd. (Wuhan, China). The PCR amplification was performed in a 50 µL reaction system containing 5 µL of 10× PCR Buffer, 1 µL of dNTPs (10 µmol/L), 1 µL of template DNA, 1 µL each of forward and reverse primers, 0.5 µL of rTaq DNA polymerase (2 U/µL), and 40.5 µL of ddH₂O. The amplification protocol was set as follows: initial denaturation at 94 °C for 5 min; 35 cycles of denaturation at 94 °C for 30 s, annealing at 55 °C for 30 s, and extension at 72 °C for 1 min 20 s; followed by a final extension at 72 °C for 10 min. The PCR products were verified by 1% agarose gel electrophoresis and then sent to Tsingke Biotech Co., Ltd. (Wuhan, China) for Sanger sequencing. The sequencing chromatograms were analyzed with Mutation Surveyor software, and genotypes were determined by alignment with the NCBI reference sequence (GenBank Accession No.: NG_012088.1). Strict quality control measures were implemented throughout the genotyping process: (1) Detection success rate: Genomic DNA extraction and Sanger sequencing were successfully completed for all 70 samples (35 cases and 35 controls), achieving a 100% genotyping success rate. No samples were excluded due to extraction failure or sequencing ambiguity. (2) Validation steps: To verify genotyping accuracy, 20% of the samples (14 samples: 7 cases and 7 controls) were randomly selected for repeat Sanger sequencing by an independent laboratory technician. The repeat results were fully concordant with the original findings, confirming the reliability of the genotyping data. (3) Blinding procedures: A single-blind design was adopted throughout the genotyping process. Laboratory personnel responsible for DNA extraction, PCR amplification, and sequencing analysis were provided only with unique sample codes, with no access to case–control status, clinical data, or IL-10 expression levels. Group assignments were disclosed only after all genotyping results had been determined and recorded, thereby minimizing the risk of subjective bias.

### Statistical analysis

Data were analyzed using SPSS 23.0. Continuous data were assessed for normality and homogeneity of variance. Normally distributed data are presented as mean ± standard deviation (*x̄* ± s) and compared between groups with the independent samples *t*-test. Non-normally distributed data are expressed as median (interquartile range) [*M* (Q1, Q3)]and compared using the Mann-Whitney *U* test. Allele frequencies were determined by direct counting. Hardy-Weinberg equilibrium for genotype distributions, as well as group comparisons of genotypes and alleles were performed using the *χ²* test or Fisher’s exact test, as appropriate. A *P*-value of < 0.05 was considered statistically significant for all tests. No correction for multiple testing was performed; thus, the nominal *P*-values should be interpreted with caution.

### Sample size and power calculation

Sample size and statistical power calculations for this genetic association study were conducted using G*Power 3.1 software (Heinrich-Heine-Universität Düsseldorf, Germany). The chi-square test model was applied to evaluate allelic and genotypic associations in a 1:1 case-control design. A two-tailed significance level (α) of 0.05 and a target power (1-β) of 0.80 were used, in line with the generally accepted standard in the field. The expected effect size, expressed as an odds ratio (OR) of 1.8, was estimated based on previous studies of cytokine gene polymorphisms. Minor allele frequencies for the analyzed IL-10 polymorphisms were derived from the control group of the present study: 8.57% for the G allele of -1082 A/G, 27.14% for the C allele of -819T/C, and 27.14% for the C allele of -592 A/C. Prospective sample size estimation indicated that achieving 80% statistical power would require 368 cases and 368 controls for the − 1082 A/G locus, and 92 cases and 92 controls for the − 819T/C and − 592 A/C loci. Retrospective power analysis showed that with the actual sample size (35 cases and 35 controls), the statistical power was only 6.2% for the − 1082 A/G locus and 26.8% for the − 819T/C and − 592 A/C loci, which are substantially below the recommended threshold.

## Results

### Inflammatory markers and plasma IL-10 Levels

The acute-phase inflammatory marker profiles of the 35 pediatric HNL patients are summarized in Table 1, which demonstrates the typical laboratory features of HNL: decreased peripheral WBC, elevated ESR, and normal CRP and PCT levels. All plasma samples for IL-10 detection were collected during the acute phase of HNL to ensure that cytokine levels reflected the active inflammatory state. The plasma IL-10 level was 5.89 (3.87, 8.69) pg/mL in the case group, compared to 3.73 (2.78, 4.24) pg/mL in the control group. This difference was statistically significant (Z = -3.338, *P* = 0.001).

**Table 1:** Inflammatory markers of HNL patients

Inflammatory marker	Results [M (Q1, Q3)]	Normal range
WBC	3.79 (3.00, 4.21)	4.3–11.3 (×10^9^∙L^− 1^)
ESR	27.00 (19.00, 36.50)	male 0–15 (mm∙H^− 1^) female 0–20 (mm∙H^− 1^)
CRP	5.20 (3.10, 8.80)	< 10 (mg∙L^− 1^)
PCT	0.15 (0.09, 0.22)	< 0.50 (ng∙mL^− 1^)

### IL-10 gene promoter polymorphisms

Genotype distributions of the IL-10 gene polymorphic loci conformed to the Hardy-Weinberg equilibrium (HWE) in both the case and control cohorts, with all *P*-values exceeding 0.05 (IL-10–1082 A/G: case group, χ²=0.21, *P* = 0.65; control group, χ²=0.91, *P* = 0.11; -819T/C and − 592 A/C: case group, χ²=0.96, *P* = 0.33 for each locus; control group, χ²=1.48, *P* = 0.22 for each locus). This result demonstrated that the study samples were representative of the Han Chinese population. Notably, only two genotypes were observed at the − 1082 A/G locus in the case group, leading to very small expected cell counts. This warrants cautious interpretation of the HWE estimate for this locus, even though a non-significant *P*-value indicated that the locus was in HWE. Two alleles were detected at each of the three IL-10 promoter loci (-1082 A/G, -819T/C, -592 A/C) in both groups. At the − 1082 locus, two genotypes were detected in the case group, whereas three genotypes were observed at both the − 819 and − 592 loci in this group. In contrast, all three loci each yielded three genotypes in the control group. Further analysis indicated that there were no statistically significant differences in allele frequencies or genotype distributions at any of the loci between the two groups (*P* > 0.05). Detailed results are presented in Tables 2 and 3.

**Table 2:** Comparison of IL-10 -1082, -819, and -592 allele frequencies between groups

Gene Locus	Allele		Case Group (*n* = 35)	Control Group (*n* = 35)	χ²	*P* Value	OR (95%CI)
-1082 A/G		A	65(92.86%)	64(91.43%)	0.10	0.75	1.22 (0.35–4.20)
		G	5(7.14%)	6(8.57%)			
-819T/C		T	53(75.71%)	51(72.86%)	0.15	0.70	1.16 (0.54–2.48)
		C	17(24.29%)	19(27.14%)			
-592 A/C		A	53(75.71%)	51(72.86%)	0.15	0.70	1.16 (0.54–2.48)
		C	17(24.29%)	19(27.14%)			

*CI* confidence interval

**Table 3:** Comparison of IL-10 -1082, -819, and -592 genotypes between groups

Gene Locus	Genotype		Case Group (*n* = 35)	Control Group (*n* = 35)	*P* Value	OR (95%CI)
-1082 A/G		AA	30 (85.71%)	30 (85.71%)	1.00	1.00(0.26–3.82)
		AG	5 (14.29%)	4 (11.43%)		
		GG	0	1 (2.86%)		
-819T/C		TT	19 (54.28%)	20 (57.14%)	0.82	1.13(0.45–2.82)
		TC	15 (42.86%)	11 (31.43%)		
		CC	1 (2.86%)	4 (11.43%)		
-592 A/C		AA	19 (54.28%)	20 (57.14%)	0.82	1.13(0.45–2.82)
		AC	15 (42.86%)	11 (31.43%)		
		CC	1(2.86%)	4(11.43%)		

Fisher’s exact test (two-tailed, α = 0.05) was used to analyze 2 × 2 contingency tables constructed under a dominant genetic model. For each locus, the homozygous wild-type genotype was designated as the reference (AA for − 1082 A/G and − 592 A/C; TT for − 819T/C), while the combined heterozygous and homozygous variant genotypes (AG + GG for − 1082 A/G; TC + CC for − 819T/C; AC + CC for − 592 A/C) were defined as the variant group

Furthermore, haplotype analysis of the -1082, -819, and -592 SNPs was performed using specialized software [8], which reconstructed three possible haplotypes. The frequency distribution of these haplotypes was comparable between the two groups, with no statistically significant difference (*P* > 0.05) (Table 4).

**Table 4:** Comparison of IL-10 haplotypes between groups

Haplotype	Case(freq)	Control(freq)	χ²	*P* Value	OR (95%CI)
G C C	5.00(7.14%)	6.00(8.57%)	0.10	0.75	0.82 (0.24–2.82)
A T A	53.00(75.71%)	51.00(72.86%)	0.15	0.70	1.16 (0.54–2.48)
A C C	12.00(17.14%)	13.00(18.57%)	0.05	0.83	0.91 (0.38–2.16)

## Discussion

HNL is a benign, immune-related lymphadenopathy of unclear etiology. Its pathogenesis is thought to involve genetic susceptibility and immune dysregulation triggered by exogenous stimuli. Clinically, the acute phase is well-characterized by leukopenia, elevated ESR, normal or mildly elevated CRP, and normal PCT levels [1, 9]. The pediatric patients with HNL enrolled in this study consistently exhibited these classic features, consistent with the non-infectious inflammatory nature of the disease. Given the pivotal role of the cytokine IL-10 in regulating and resolving inflammation, we sought to preliminarily investigate its expression levels and gene polymorphism profiles in this patient cohort.

The results of this study showed that the plasma IL-10 levels were significantly increased in children with HNL during the acute phase with typical inflammatory manifestations. This finding suggests that the upregulation of IL-10 may be a compensatory anti-inflammatory response of the body to the abnormal immune activation in HNL, which attempts to negatively regulate the excessive inflammatory process, and is consistent with the biological function of IL-10 as a classic anti-inflammatory cytokine [3]. Previous studies have shown that genetic variants in the IL-10 promoter influence IL-10 production. Specifically, Gallagher et al. [10] reported that the − 1082G allele is associated with high IL-10 expression, whereas the − 1082 A allele correlates with low expression. Sainz et al. [11] demonstrated that the − 592 C allele corresponds to higher IL-10 expression compared to the − 592 A allele. Moreover, Oduor et al. [12] identified that the GCC haplotype formed by these three loci is associated with high IL-10 expression, while the ATA haplotype is linked to low expression. In the present study, however, no statistically significant differences were identified in the genotype, allele, and haplotype distributions of the three IL-10 promoter SNPs between the case and control groups. This limited sample size stemmed from the low incidence of pediatric HNL, resulting in the study’s statistical power being substantially below the widely recommended 80% threshold (6.2% for the − 1082 A/G locus and 26.8% for the − 819T/C and − 592 A/C loci). As such, these results should be construed as exploratory rather than conclusive, and a potential association of IL-10 polymorphisms with susceptibility to pediatric HNL cannot be excluded.

In conclusion, to our knowledge, this is the first study to report significantly elevated plasma IL-10 levels in pediatric patients with HNL who presented with typical acute-phase clinical and laboratory features. Our findings suggest that IL-10-mediated immune dysregulation may play a critical role in the pathogenesis of HNL, thereby providing a novel direction for future mechanistic investigations. No significant associations were found between the three IL-10 promoter polymorphisms (-1082 A/G, -819T/C, -592 A/C) and pediatric HNL susceptibility. These findings should be considered exploratory, owing to the limited sample size and consequent insufficient statistical power resulting from the low incidence of the disease.

This study has several limitations that should be acknowledged. First, the small sample size due to the rarity of pediatric HNL limits statistical power, so the genetic findings are preliminary and require validation in larger, multicenter cohorts. Second, as a single-center study restricted to Han Chinese children, the generalizability of our findings to other ethnic populations may be limited. Third, only three common SNPs in the IL-10 promoter were analyzed; other functional variants or epigenetic modifications were not investigated. Fourth, no multiple testing correction was made, which may increase the risk of Type I error. Future studies with larger, multi-ethnic cohorts and comprehensive genetic and epigenetic analyses are needed to validate these findings and further elucidate the role of IL-10 in pediatric HNL.

## Data Availability

All data generated or analyzed during this study are included in this published article and its supplementary information files.

## Abbreviations

IL-10: interleukin-10
HNL: Histiocytic Necrotizing Lymphadenitis
SNPs: Single Nucleotide Polymorphisms
SLE: Systemic Lupus Erythematosus
IBD: Inflammatory Bowel Disease
WBC: Peripheral White Blood Cell
ESR: Erythrocyte Sedimentation Rate
CRP: C-reactive Protein
PCT: Procalcitonin
ELISA: Enzyme-linked Immunosorbent Assay
CV: Coefficient of Variation
QC: Quality Control
OR: Odds Ratio
HWE: Hardy-Weinberg equilibrium
CI: Confidence Interval
